# Outcome of Surgical Treatment in Late-Onset Capsular Block Syndrome

**DOI:** 10.1155/2017/1847179

**Published:** 2017-07-09

**Authors:** Yang Huang, Zi Ye, Hang Li, Zhaohui Li

**Affiliations:** ^1^Department of Ophthalmology, The Chinese People's Liberation Army General Hospital, Beijing, China; ^2^Medical Department, The First Hospital Affiliated to General Hospital of the Chinese People's Liberation Army, Beijing, China

## Abstract

**Purpose:**

To further investigate the pathogenesis of late-onset capsular block syndrome (CBS) and to evaluate the safety of surgical treatment.

**Methods:**

Seven patients diagnosed with late-onset CBS were retrospectively analyzed. Anterior chamber depth (ACD), intraocular pressure (IOP), refractive diopter, and best-corrected visual acuity (BCVA) before and after surgery were recorded. The opaque substance was tested with Western blot, and a flow cytometer multiple array assay system was utilized to evaluate the levels of inflammatory cytokines from opaque substance and aqueous humor, respectively.

**Results:**

Patients who had undergone surgical treatment showed a significant BCVA and spherical equivalent refractive error improvement (*P* = 0.002, *P* = 0.021, resp.). Nevertheless, ACD and IOP before and after surgery were in normal range with no difference (*P* = 0.165, *P* = 0.749, resp.). *α*B-crystallin and *β*B-crystallin were detected in all opaque substances. Tumor necrosis factor-alpha (TNF-*α*) and interlukin-1*β* (IL-1*β*) levels in opaque substance were significantly higher than those in aqueous humor (*P* = 0.038, *P* = 0.007, resp.), while IL-2 and IL-6 were not detected in any samples.

**Conclusions:**

Opaque substance is derived from human lens epithelial cells. Inflammatory cytokines may be involved in the pathogenesis of late-onset CBS. In addition, surgical treatment is an effective approach. This trial is registered with ChiCTR-IOR-17011287.

## 1. Introduction

Capsular block syndrome (CBS) is a rare complication related to phacoemulsification, which is described as the accumulation of opaque substance between the posterior chamber intraocular lens (PC-IOL) and posterior capsule, and was first described by Davison [1, 2]. Based on the time of onset, Miyake et al. [3] classified CBS as intraoperative (caused by high irrigation pressure during hydrodissection maneuvers), early postoperative (due to osmotic gradient accumulation), and late-onset (originated from residual lens epithelial cells).

A variety of clinical manifestation of early postoperative CBS include a shallow anterior chamber, an unexpected myopic shift, or elevation of intraocular pressure (IOP) [4]. Nevertheless, late-onset CBS lack these features and may not be recognized until best-corrected visual acuity (BCVA) was compromised to a certain extent [5].

Some reports have investigated the pathogenesis of late-onset CBS. Eifrig first confirmed that opaque substance derives from residual lens epithelial cells (LECs) for relatively high alpha-crystallin and low albumin levels [6]. Bao then shared the same results via analyzing milky liquid spectrometrically [7]. In addition, blood-aqueous barrier malfunctions were also recognized. Increased aqueous flare intensity and cells were observed in more than half of these patients [8], while the relation of inflammatory cytokines levels from opaque substance and aqueous humor in late-onset CBS has yet to be recognized.

Neodymium:yttrium-aluminum-garnet (Nd:YAG) laser capsulotomy has become the major approach to cure late-onset CBS for its convenience and microinvasion [9]. However, several severe complications, especially *P. acnes*-associated endophthalmitis, still exist [10, 11]. Sometimes, dense opaque substance and severe expansion of the posterior capsule make laser capsulotomy difficult [12]. Thus, surgical intervention seems to be a reliable alternative. In this report, we aimed to further investigate inflammatory cytokine levels in the pathogenesis of late-onset CBS and evaluate the safety of surgical treatment.

## 2. Materials and Methods

This study was approved by the Institutional Review Board and Ethics Committee of the Chinese People's Liberation Army General Hospital, and informed consent was obtained from all participants.

### 2.1. Study Participants

We collected cases of late-onset CBS in patients diagnosed and treated in the Chinese People's Liberation Army General Hospital from July 2014 to July 2016 retrospectively. Late-onset CBS was identified by slit-lamp examination as opaque substance accumulated between the posterior capsule and posterior surface of PC-IOL more than one year after cataract surgery. According to reports published by Wisconsin epidemiologic study of diabetic retinopathy (WESDR), diabetes mellitus was diagnosed [13].

### 2.2. Measurement of Parameters

All biometric parameters were measured within 1 week before and 3 months after surgery. ACD was measured using a Pentacam (HR70900, OCULUS Optikgerate Gmbh, Wetzlar, Germany) with 3 continuous anterior chamber images. The measurement was repeated on another day by the same technician and two results were averaged. IOP was recorded as the mean of 3 values using a noncontact tonometer (NCT; TX-F, Canon Inc., Tokyo, Japan). An abnormal IOP was defined as 21 mmHg or higher. The refractive diopter before and after surgery was recorded using an autofractometer (KR-8100; Topcon Corporation, Tokyo, Japan). BCVA was assessed preoperatively and 3 months postoperatively and then converted into logarithm of the minimum angle of resolution (logMAR) for analysis. Fundus evaluation was detected with an indirect ophthalmoscope. All previous surgical histories and preoperative complications were identified from the patients' medical records.

### 2.3. Surgical Approach

All surgeries were conducted by one single physician. Prior to operation, tropicamide eye drops were administered for full mydriasis and oxybuprocaine for topical anesthesia. Aqueous humor sample was obtained through anterior chamber paracentesis using a 30 G needle on a 1 mL syringe; then, a transparent tunnel 2.4 mm in diameter was made in the upper cornea and 1% sodium hyaluronate was injected to maintain the anterior chamber. Through the corneal tunnel, a 30 G needle on a 1 mL syringe was used to penetrate the anterior capsule and aspirate the opaque substance between the posterior capsule and posterior surface of PC-IOL. Next, Vannas scissors were utilized to remove all fibrotic tissue on the anterior capsule and enlarge anterior capsular opening. Then, irrigation/aspiration (I/A) was performed to polish the posterior capsule and to flush the entire capsular bag. After viscoelastic material was aspirated, the anterior chamber was formed using balanced solution. Aqueous humor and opaque substance (0.1–0.2 ml) were collected in sterile tubes and frozen to −80°C until further analysis, respectively.

### 2.4. Western Blot Analysis of Opaque Substance

A BCA assay kit (CoWin Biotech, Beijing, China) was used to detect the protein concentration of opaque substance. Protein was added to 10% SDS-polyacrylamide gels, transferred to polyvinylidene difluoride (PVDF) membranes, and probed with antibodies against *α*B-crystallin (1 : 1000) and *β*B-crystallin (1 : 1000) (Abcam, Cambridge, UK). Then, HRP-conjugated secondary antibody was incubated. The ECL chemiluminescence system (Pierce Biotechnology, USA) was for blot detection. Finally, we utilized ImageJ software (v1.40) to analyze the protein bands.

### 2.5. Inflammatory Cytokine Analysis of Opaque Substance

Measurement of inflammatory cytokines (TNF-*α*, IL-1*β*, IL-2, and IL-6) in aqueous humor and opaque substance was performed employing a Luminex®200™ flow cytometer (Merck Millipore, Germany) multiple array assay system using Multiplex kits (Millipore) based on the Luminex xMAP technology designed to spontaneously measure multiple protein targets in a single sample at a very limited assay volume according to the manufacturer's instruction. The MILLIPLEX MAP Human Cytokine/Chemokine panel (EMD Millipore Corporation, Billerica, MA, USA) incubating 25 *μ*l of the sample with antibodies at −4°C was used. Values were expressed as pg/ml, and the standard curve range from 3.2 to 10,000 pg/ml.

### 2.6. Statistical Analysis

Data were analyzed with SPSS version 19 software and expressed as the mean ± SD. Differences between groups were analyzed by the Wilcoxon signed-rank test or the Mann–Whitney test as appropriate. *P* < 0.05 was considered statistically significant.

## 3. Results

**Figure 1:**
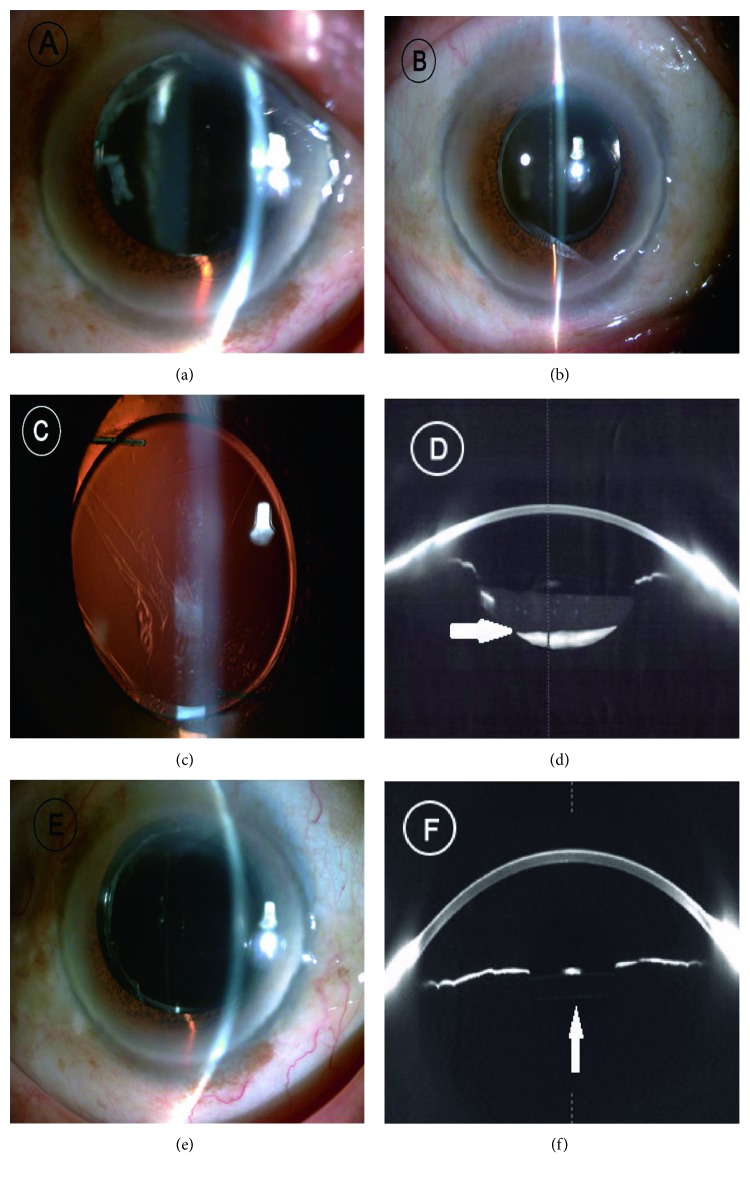
(a–c) Slit-lamp microscopy showed fibrotic anterior capsule opening and PCO formation in late-onset CBS. (d) The high density of the opaque substance between the posterior surface of PC-IOL and posterior capsule was verified via Scheimpflug photograph. White arrow indicated the opaque substance. (e, f) After surgical treatment, the opaque substance vanished and the posterior capsule was attached to the posterior surface of PC-IOL. White arrow showed the normal anterior chamber.

The clinical data from 7 eyes of 7 patients who were diagnosed with late-onset CBS was established in Table 1. All patients were of diabetes mellitus. They disclosed a distension of the posterior capsular bag with opaque substance accumulation. Obviously fibrotic anterior capsular opening and mild posterior capsule opacification (PCO) were also observed (Figures 1(a), 1(b), 1(c), and 1(d)). In addition, no diabetic retinopathy was found via fundus examination. After surgical treatment, the intracapsular fluid turned to be clear, the posterior capsule was attached to the posterior surface of PC-IOL, and residual LECs were removed (Figures 1(e) and 1(f)).

**Figure 2:**
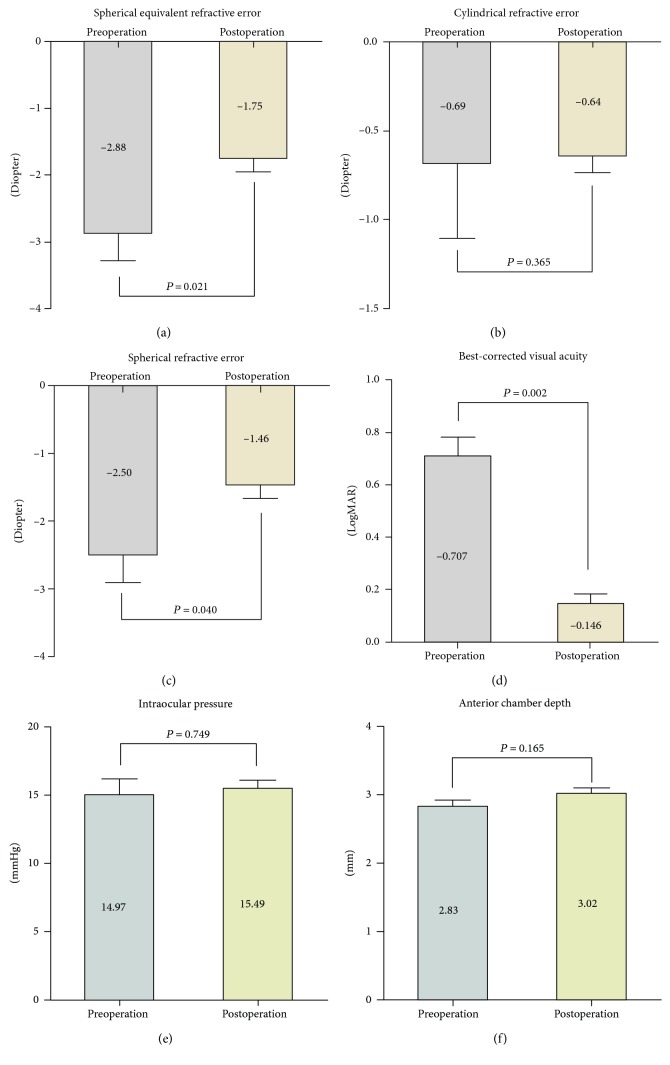
(a–c) The averaged refractive error in late-onset CBS was displayed with 95% confidence interval. Each test was done 1 week before surgical intervention and 3 months after surgical intervention. (d) BCVA after surgical treatment improved in all patients in LogMAR (*P* = 0.002). (e, f) IOP and ACD before and after surgical intervention showed no statistical difference (*P* = 0.749 and *P* = 0.165, resp.).

All patients completed a 3-month follow-up. The mean interval between phacoemulsification and surgical intervention was 37.43 ± 13.48 months (mean ± SD, range, 15 to 53 months). The spherical equivalent refractive error after surgical intervention changed from −2.88 ± 1.09 diopter (D) (mean ± SD, range, −5.25 to −1.75 D) to −1.75 ± 0.53 D (mean ± SD, range, −2.50 to −0.75 D) (Figures 2(a), 2(b), and 2(c)); thus, a reduction in myopia was noticed (+1.125 ± 0.736 D, range, +0.25 D to +2.625 D). BCVA improved in all patients (from 0.707 ± 0.192 to 0.146 ± 0.094 (mean ± SD), in LogMAR, *P* = 0.002) (Figure 2(d)). The IOP before and after surgical treatment were in normal range with no difference (*P* = 0.749) (Figures 2(e) and 2(f)); the topical glaucoma medication was not used in any cases. The ACD before and after surgical treatment showed no change (*P* = 0.165); PC-IOL remained in situ. Severe complications such as anterior chamber inflammation, cystoid macular edema, or retinal detachment were not proved.

**Figure 3:**
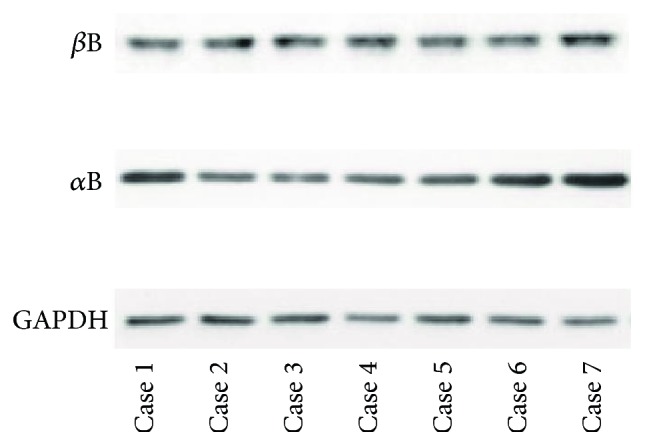
Western blot analysis of opaque substance. High concentrations of *α*B- and *β*B-crystallin were confirmed in all cases.

As the data showed (Figure 3), *α*B-crystallin and *β*B-crystallin were detected in all opaque substances by Western blot.

**Figure 4:**
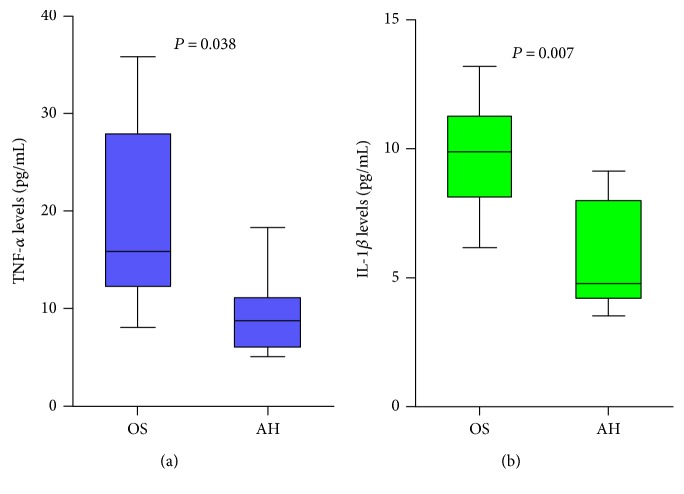
(a, b) The TNF-*α* and IL-1*β* levels in opaque substance were higher compared to those in aqueous humor (*P* = 0.038, *P* = 0.07, resp.), while IL-2 and IL-6 were not detected in any samples.

Inflammatory cytokine levels in aqueous humor and opaque substance were tested via the flow cytometer multiple array assay system (Figures 4(a) and 4(b)). TNF-*α* and IL-1*β* levels were detected in all patients, while IL-2 and IL-6 were not verified in any samples. The TNF-*α* levels in opaque substance (18.68 ± 9.76 pg/ml, range, 8.11 to 35.77 pg/ml) compared to those in aqueous humor (9.37 ± 4.39 pg/ml, range, 5.11 to 18.25 pg/ml) were higher (*P* = 0.038). The IL-1*β* levels in opaque substance (9.56 ± 2.29 pg/ml, range, 6.18 to 13.18 pg/ml) compared to those in aqueous humor (5.61 ± 2.12 pg/ml, range, 3.53 to 9.12 pg/ml) were also statistically different (*P* = 0.007).

## 4. Discussion

Recently, Nd:YAG laser treatment, including anterior and posterior laser capsulotomy, has been recommended as the major approach to cure late-onset CBS for its microinvasion and convenience [9, 14], while more and more authors are worried about the potential complications after posterior laser capsulotomy, such as vitreous floaters, cystoid macular edema, and retinal detachment [15–17]. Several reports of late-onset CBS have also been verified to be related to *Propionibacterium acnes*; hence, after posterior laser capsulotomy, *P. acnes*-associated endophthalmitis may occur [10, 11, 18, 19]. Moreover, posterior laser capsulotomy is difficult to perform because of opaque substance or severe expansion of the posterior capsule [12]. Thus, to some extent, anterior laser capsulotomy seems to be a good option. Unfortunately, the risk of IOP elevation, wrinkling of the posterior capsule, and high recurrence rate can be disadvantages [20, 21]. Colakoglu et al. demonstrated that anterior laser capsulotomy would release opaque substance containing collagen and extracellular matrix into the anterior chamber that may be difficult to drain via anterior chamber angle resulting in uncontrollable IOP elevation. Meanwhile, milky-white fluid may reform soon after such procedure [22]. Koh et al. surmised that LEC proliferation and anterior capsulotomy opening clogging may cause recurrence of late-onset CBS [23]. Thereby, on the basis of present results, we take surgical treatment into consideration.

In our patients, there were no severe complications during the follow-up period, such as uncontrollable IOP elevation, uveitis, endophthalmitis, cystoid macular edema, or retinal detachment; thus, we thought that anterior capsule release, opaque substance aspiration, and posterior capsule polishing via transparent corneal tunnel may be a safe and effective alternative. Surgical management is easy to perform, and it can (1) enlarge anterior capsular opening, preventing recurrence of CBS; (2) aspirate opaque substance and residual lens epithelial cells thoroughly, preventing occurrence of endophthalmitis and IOP elevation; and (3) maintain the integrity of the posterior capsule, preventing vitreous floater, cystoid macular edema, and retinal detachment formation.

Compared with early postoperative CBS, several studies have disclosed that late-onset CBS may be less likely to be involved in myopic shift, shallow ACD, or IOP elevation [24, 25]. Landa et al. showed a mean refractive error change < 0.50 D in patients with late-onset CBS following posterior laser capsulotomy [4]. Pinarci et al. got a similar result in 15 eyes [26]. Nevertheless, both Bao et al. and Yang et al. pointed out that significantly hyperopic changes occurred after posterior laser capsulotomy [7, 27]. In our patients, we also noticed a remarkable reduction in myopia after surgical drainage without PC-IOL displacement. This may be based on opalescent materials which act as a phakic IOL rather than forward displacement of the PC-IOL as fibrotic anterior capsule opening formation [26]. Therefore, even without IOL forward displacement, a myopic shift may occur.

In the literature, the occurrence of late-onset CBS formation is based on two major factors: integrated posterior capsule and residual LECs. First, the anterior surface of PC-IOL totally blocks anterior capsule opening that makes the capsular bag a closed space. As a semipermeable membrane, the posterior capsule then accumulates fluid into the closed capsular bag via the difference in osmotic pressure inside and outside the bag [28]. The levels of osmotic pressure have been evidenced to be the result of proteins constantly secreted from residual LECs. Eifrig et al. was the first to speculate that liquid derives from the residual LECs of the cataract for high concentration of alpha-crystallin and relatively low albumin levels in opaque substance [6]. Meanwhile, they did not detect any globulin in the liquid, which suggested no immune response is involved. Bao et al.'s biochemical analysis of the closed bag also showed that the concentrations of proteins that proved to be crystallins and crystallin peptide by mass spectrometry and Ca^2+^ were higher than the concentrations in the aqueous humor of the patient [7]. This could cause the difference in osmotic pressure between the two sides of the capsular bag. As the proteins increase, the osmotic pressure inside the capsular bag increases and more liquid accumulates. In the present study, we also proved that fibrotic anterior capsule opening and obvious PCO exist in all patients, and opaque substance contains a high concentration of *α*B-crystallin and *β*B-crystallin.

Beyond Miyake et al.'s classification of CBS, Kim et al. classified the clinical characteristics of CBS as noncellular, inflammatory, and fibrotic, which is controversial till now for lacking experimental confirmation and having rather small samples [29]. In Kim et al.'s opinion, late-onset CBS was recognized as fibrotic CBS with no cellular reaction. Nevertheless, several studies have demonstrated that late-onset CBS often occurs with enhanced aqueous flare intensity, especially in patients with diabetes mellitus, uveitis, and glaucoma, which is suspected to be related to breakdown of the blood-aqueous barrier [6, 8]. Kitagawa et al. quantitatively demonstrated high cell and protein levels in the anterior chamber of patients diagnosed with late postoperative CBS by a laser flare cell meter, which represented a direct evidence of blood-aqueous barrier malfunctions [30]. However, whether blood-aqueous barrier malfunctions are the reason or the result of late-onset CBS remains unknown. Thus, we detected the inflammatory cytokines, which can result in a breakdown of blood-aqueous barrier, in both opaque substance and aqueous humor. Preliminarily, we measured 4 important proinflammatory cytokines (TNF-*α*, IL-1*β*, IL-2, and IL-6) via the flow cytometer multiple array assay system for 3 individual experiments. Interestingly, our results uncovered that TNF-*α* and IL-1*β* were statistically higher in opaque substance, while IL-2 and IL-6 were undetectable. There is numerous evidence that TNF-*α* and IL-1*β* play a critical role in cell dysfunction. TNF-*α* can impair nitric oxide- (NO-) mediated vasodilation, increase production of reactive oxygen species (ROS), activate expression of transcription factor NF-*κ*B, and regulate both proliferation and apoptosis [31, 32]. Meanwhile, IL-1*β* can upregulate several inflammatory mediators, including IL-1*β* itself, TNF-*α*, cyclooxygenase-2 (COX-2), prostaglandins, inducible nitric oxide synthase (iNOS), and chemokines, resulting in acceleration of the apoptosis of cells via the activation of NF-*κ*B [33, 34]. This may be the rational explanation of blood-aqueous barrier malfunction formation in late-onset CBS.

However, it still made us lost in thought. Why is TNF-*α* and IL-1*β* higher in opaque substance? What is the relationship between proinflammatory cytokines (TNF-*α* and IL-1*β*) and late-onset CBS formation? Previously, Nishi et al. confirmed that interleukin-1 can be synthesized by residual LECs of human cataract [35]. Therefore, we hypothesize that residual LECs may release proinflammatory cytokines (TNF-*α* and IL-1*β*) consistently, which leads to anterior capsule opening fibrosis, and attach tightly to the anterior surface of PC-IOL; then, opaque substances accumulate and a spot of proinflammatory cytokines leak to aqueous humor, leading to blood-aqueous barrier breakdown.

In conclusion, late-onset CBS occurred long-term after cataract surgery and may not be noticed until visual acuity compromised. The recommended treatment in many studies was Nd:YAG laser capsulotomy, but several severe complications could emerge and may even cause a dissemination of intraocular infection. In the present study, we further clarified that opaque substance in the capsular bag is derived from human lens epithelial cells. Meanwhile, blood-aqueous barrier malfunctions exist and inflammatory cytokines may be involved in pathogenesis of late-onset CBS. Eventually, we also proved that late-onset CBS can be successfully treated by surgical drainage without serious complications, which we believe is a safe and effective approach.

## Figures and Tables

**Table 1:** Demographics and clinical data of all patients.

Parameter		Value
Patients		7
Eyes	Total	7
	Right : left	5 : 2
Mean age (y) ± SD		68.29 ± 6.26
Sex (M/F)		4 : 3
Onset time after phaco (m) ± SD		37.43 ± 13.48
Intraocular lens type	Hydrophobic, 1 piece	4
	Hydrophilic, 3 piece	1
	Hydrophilic, 1 piece	1
	Unknown	1

## References

[1] Davison J. A. (1990). Capsular bag distension after endophacoemulsification and posterior chamber intraocular lens implantation. *Journal of Cataract and Refractive Surgery*.

[2] Nishi O., Nishi K., Takahashi E. (1998). Capsular bag distention syndrome noted 5 years after intraocular lens implantation. *American Journal of Ophthalmology*.

[3] Miyake K., Ota I., Ichihashi S., Miyake S., Tanaka Y., Terasaki H. (1998). New classification of capsular block syndrome. *Journal of Cataract and Refractive Surgery*.

[4] Landa G., Hoffman P., Pollack A., Bukelman A., Leiba H., Marcovich A. (2006). Outcome of posterior capsulotomy in late capsular block syndrome with posterior capsular opacification. *Clinical and Experimental Ophthalmology*.

[5] Masket S. (1993). Postoperative complications of capsulorhexis. *Journal of Cataract and Refractive Surgery*.

[6] Eifrig D. E. (1997). Capsulorhexis-related lacteocrumenasia. *Journal of Cataract and Refractive Surgery*.

[7] Bao Y. Z., Pei X. T., Li M. W., Li X. X. (2008). Late postoperative capsular block syndrome versus liquefied after-cataract. *Journal of Cataract and Refractive Surgery*.

[8] Miyake K., Ota I., Miyake S., Horiguchi M. (1998). Liquefied after cataract: a complication of continuous curvilinear capsulorhexis and intraocular lens implantation in the lens capsule. *American Journal of Ophthalmology*.

[9] Grover D. S., Goldberg R. A., Ayres B., Fantes F. (2012). Treatment of late-onset capsular distension syndrome with a neodymium:YAG laser peripheral iridotomy and anterior capsulotomy. *Journal of Cataract and Refractive Surgery*.

[10] Kollias A. N., Vogel M. A., de Kaspar H. M., Lackerbauer C. A., Grueterich M. (2010). Propionibacterium acnes in capsular bag distension syndrome. *Journal of Cataract and Refractive Surgery*.

[11] Carlson A. N., Koch D. D. (1988). Endophthalmitis following Nd:YAG laser posterior capsulotomy. *Ophthalmic Surgery*.

[12] Qu J., Bao Y., Li M., Zhao M., Li X. (2010). Surgical management of late capsular block syndrome. *Journal of Cataract and Refractive Surgery*.

[13] Varma R. (2008). From a population to patients: the Wisconsin epidemiologic study of diabetic retinopathy. *Ophthalmology*.

[14] Pinsard L., Rougier M. B., Colin J. (2011). Neodymium:YAG laser treatment of late capsular block syndrome. *Journal of Cataract and Refractive Surgery*.

[15] Sorenson A. L., Holladay J. T., Kim T., Kendall C. J., Carlson A. N. (2000). Ultrasonographic measurement of induced myopia associated with capsular bag distention syndrome. *Ophthalmology*.

[16] Holtz S. J. (1992). Postoperative capsular bag distension. *Journal of Cataract and Refractive Surgery*.

[17] Durak I., Ozbek Z., Ferliel S. T., Oner F. H., Söylev M. (2001). Early postoperative capsular block syndrome. *Journal of Cataract and Refractive Surgery*.

[18] Dhaliwal D. K., Farhi P., Eller A. W., Kowalski R. P. (2011). Late capsular block syndrome associated with Propionibacterium acnes. *Archives of Ophthalmology*.

[19] Rana M., Jiang L., Ilango B., Yang Y. C. (2013). Late-onset capsular block syndrome: unusually delayed presentation. *Case Reports in Ophthalmology*.

[20] Durig J., Zografos L. (1999). Posterior capsule opacification and wrinkling in a case of capsular bag distension. *European Journal of Ophthalmology*.

[21] Shah N. A., Goulstine D. B. (2006). Capsular block syndrome presenting with a hyperopic shift. *Journal of Cataract and Refractive Surgery*.

[22] Colakoglu A., Kucukakyuz N., Topcuoglu I. E., Akar S. (2007). Intraocular pressure rise and recurrence of capsular block syndrome after neodymium: YAG laser anterior capsulotomy. *Journal of Cataract and Refractive Surgery*.

[23] Koh J. S., Song Y. B., Wee W. R., Han Y. K. (2016). Recurrent late-onset fibrotic capsular block syndrome after neodymium-yttrium-aluminum-garnet laser anterior capsulotomy: a case report. *BMC Ophthalmology*.

[24] Omar O., Eng C. T., Chang A., Durcan F. J., Liss R. P., Stark B. I. (1996). Capsular bag distension with an acrylic intraocular lens. *Journal of Cataract and Refractive Surgery*.

[25] Zhu X. J., Zhang K. K., Yang J., Ye H. F., Lu Y. (2014). Scheimpflug imaging of ultra-late postoperative capsular block syndrome. *Eye (London, England)*.

[26] Pinarci E. Y., Bayar S. A., Sizmaz S., Canan H., Yilmaz G. (2012). Late capsular block syndrome presenting with posterior capsule opacification. *Journal of Cataract and Refractive Surgery*.

[27] Yang M. K., Wee W. R., Kwon J. W., Han Y. K. (2015). Anterior chamber depth and refractive change in late postoperative capsular bag distension syndrome: a retrospective analysis. *PLoS One*.

[28] Theng J. T., Jap A., Chee S. P. (2000). Capsular block syndrome: a case series. *Journal of Cataract and Refractive Surgery*.

[29] Kim H. K., Shin J. P. (2008). Capsular block syndrome after cataract surgery: clinical analysis and classification. *Journal of Cataract and Refractive Surgery*.

[30] Kitagawa K., Hayasaka S., Nagaki Y. (2004). Increased aqueous flare intensity in eyes with liquefied after-cataract. *Journal of Cataract and Refractive Surgery*.

[31] Bertazza L., Mocellin S. (2008). Tumor necrosis factor (TNF) biology and cell death. *Frontiers in Bioscience*.

[32] Gustavsson C., Agardh C. D., Agardh E. (2013). Profile of intraocular tumour necrosis factor-α and interleukin-6 in diabetic subjects with different degrees of diabetic retinopathy. *Acta Ophthalmologica*.

[33] Rothwell N. J., Luheshi G. N. (2000). Interleukin 1 in the brain: biology, pathology and therapeutic target. *Trends in Neurosciences*.

[34] Kowluru R. A., Odenbach S. (2004). Role of interleukin-1beta in the pathogenesis of diabetic retinopathy. *The British Journal of Ophthalmology*.

[35] Nishi O., Nishi K., Imanishi M. (1992). Synthesis of interleukin-1 and prostaglandin E2 by lens epithelial cells of human cataracts. *The British Journal of Ophthalmology*.

